# Giant lipoma of the adrenal gland: a case report

**DOI:** 10.1186/1752-1947-5-78

**Published:** 2011-02-24

**Authors:** Rashmi D Patel, Aruna V Vanikar, Pranjal R Modi

**Affiliations:** 1 Department of Pathology, Laboratory Medicine, Transfusion Services and Immunohematology, G.R. Doshi and K.M. Mehta Institute of Kidney Diseases and Research Centre and Dr. H.L. Trivedi Institute of Transplantation Sciences, Ahmedabad, India; 2 Department of Urology and Transplantation, G.R. Doshi and K.M. Mehta Institute of Kidney Diseases and Research Centre and Dr. H.L. Trivedi Institute of Transplantation Sciences, Ahmedabad, India

## Abstract

**Introduction:**

Lipoma of the adrenal gland is rare with a reported incidence of between 2% to 4%. Improved imaging techniques have helped in the diagnosis of these lesions.

**Case presentation:**

We report an incidentally detected giant adrenal lipoma in a 43-year-old Asian man with a six year history of hypertension. He had a myocardial infarction one year earlier, for which he was taking an antiplatelet agent in addition to antihypertensive medication.

The tumor was detected by computed tomography and magnetic resonance imaging, and was a large, well-defined, altered signal intensity lesion 12 cm in size in the right suprarenal region. The tumor was resected laparoscopically and sent for histopathologic evaluation. It measured 15 cm × 11.5 cm × 6.5 cm on gross examination, weighed 810 g and had a homogenous yellow cut surface. The postoperative course was smooth. Microscopy revealed mature adipose tissue with myxoid degeneration. Over the course of a four month follow-up the patient recovered.

**Conclusion:**

Giant lipoma of the adrenal gland, a benign tumor, is rare compared with myelolipoma. Improved radiologic modalities have led to increased reporting of these benign tumors. Laparoscopic removal of the tumor has helped in early recovery and in reinstating patients to normal lives.

## Introduction

Lipomatous tumors of the adrenal gland are rare. Improved diagnostic modalities, including high-resolution ultrasonography, computed tomography (CT) and magnetic resonance imaging (MRI), have led to increased reporting and management of such tumors. We report one such case of an incidentally detected adrenal lipoma.

## Case presentation

A 43-year-old Asian man was incidentally detected to have a loose, well-defined, homogenously enhancing soft tissue density lesion in the right adrenal gland. This lesion was found while the patient was undergoing investigations for hypertension of six years' duration being controlled with α- and β-blockers and a diuretic. The patient had a history of myocardial infarction nine earlier for which he underwent coronary angioplasty and he was taking an antiplatelet agent.

During this admission, he had undergone ultrasonography of the abdomen, which revealed an adrenal mass. No other significant history or findings were noted.

**Figure 1:**
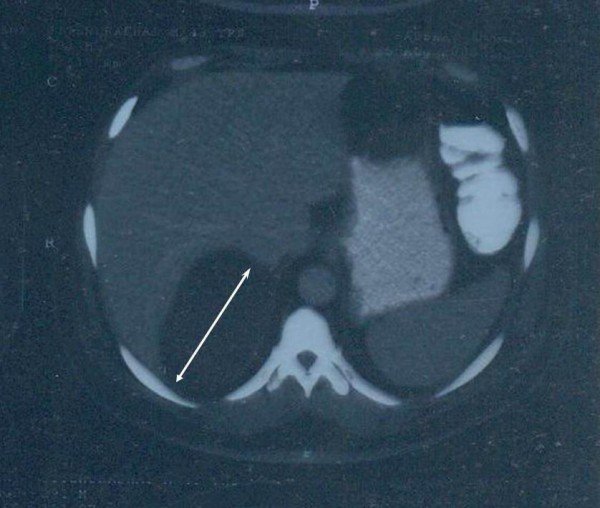
**Computed tomography scan showing a large, well-defined, soft tissue density mass in the right suprarenal region (bidirectional arrow)**.

**Figure 2:**
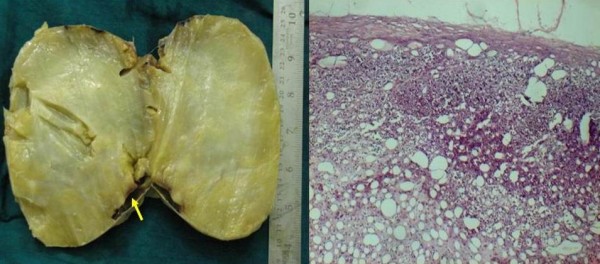
**A) Cut surface of the giant adrenal lipoma with golden yellow-tan color with an arrow showing the uninvolved adrenal gland**. B) Photomicrograph (hematoxylin and eosin stain) showing the tumor made up of mature adipose tissue intermingled with normal adrenal cortical parenchyma.

On examination, the patient's blood pressure was 150/90 mm Hg. A chest radiograph revealed prominent vascular markings in bilateral lung fields with cardiomegaly. MRI suggested adrenal myolipoma with a large, well-defined, altered signal intensity lesion in the right suprarenal region measuring 119 × 105 mm in size. Other organs were unremarkable. No lymph node enlargement was noted. CT scan revealed a large, well-defined, soft tissue density lesion with intrinsic fat density areas in the right adrenal region measuring 116 × 100 mm in size (Figure 1). On laboratory investigations, the patient's random blood sugar was 82.6 mg/dL and his renal and liver functions and electrolytes were within normal range. The patient's hemoglobin was 10.6 g/dL, and counts were within normal range and were nonreactive to HIV, hepatitis B surface antigen and hepatitis C virus on enzyme-linked immunosorbent assay. Urine analysis and microscopic findings were unremarkable. His 24-hour urinary vanillylmandelic acid level was 4.7 mg (reference range, 2-8 mg/24 h). The patient was subjected to transperitoneal laparoscopic adrenalectomy. On gross examination, a well-encapsulated tumor mass weighing 810 g measured 15 × 11.5 × 6.5 cm in size (Figure 2A). The external surface was smooth and grey-yellow colored, and the adjacent adrenal gland measuring 6 × 1.5 × 0.5 cm in size was unremarkable. The cut surface was homogenous, yellow and fatty with myxomatous areas or a jelly-like appearance and soft to firm in consistency. Microscopy revealed a well-encapsulated tumor composed of mature adipose tissue with myxoid degenerative changes and normal adrenal parenchyma (Figure 2B).

The patient had an uneventful postoperative course and is stable with the same medications continued for one month. The patient is now taking a beta-blocker and antiplatelet agents, and he has stopped taking the alpha-blocker and diuretic that he needed previously for blood pressure control.

## Discussion

Adrenal lipomas are benign tumors with a reported incidence of 2% to 4% of all adrenal tumors [1-4]. The differential diagnoses include myelolipoma, angiomyolipoma, liposarcoma and teratoma. The histogenesis of these mesenchymal tumors is still little understood. Lipomas are known to occur on the right side with male predominance as opposed to myelolipomas, which have no gender or site predilection. The size of these tumors is usually smaller than 4 cm; however, the term *giant lipoma *is preferred when the size exceeds 8 cm. Our patient had a tumor of more than 11 cm in diameter, thus falling in the category of "giant adrenal lipoma." No calcification was noted despite the lipoma's large size.

## Conclusion

Giant adrenal lipomas are rare but are being reported more frequently because of improved modern imaging technologies. This tumor was removed with the rarely reported technique of transperitoneal laparoscopic adrenalectomy and was confirmed histologically.

## Competing interests

The authors declare that they have no competing interests.

## Consent

Written informed consent was obtained from the patient for publication of this case report and accompanying images. A copy of the written consent is available for review by the Editor-in-Chief of this journal.

## Authors' contributions

RDP was the major contributor in writing the manuscript and analysis and interpretation of patient data regarding the histopathologic disease. AVV performed the histologic examination and helped in preparation of the final manuscript. PRM did the transperitoneal laparoscopic adrenalectomy and postoperative follow-up of the patient. All authors read and approved the final manuscript.
